# Layer-specificity in the effects of attention and working memory on activity in primary visual cortex

**DOI:** 10.1038/ncomms13804

**Published:** 2017-01-05

**Authors:** Timo van Kerkoerle, Matthew W. Self, Pieter R. Roelfsema

**Affiliations:** 1 Cognitive Neuroimaging Unit, CEA DSV/I2BM, INSERM, Université Paris-Sud, Université Paris-Saclay, NeuroSpin Center, Gif/Yvette 91191, France; 2 Department of Vision & Cognition, Netherlands Institute for Neurosciences, Meibergdreef 47, Amsterdam 1105 BA, The Netherlands; 3 Department of Integrative Neurophysiology, Center for Neurogenomics and Cognitive Research, VU University, Amsterdam 1081 HV, The Netherlands; 4 Psychiatry Department, Academic Medical Center, 1105 AZ Amsterdam, The Netherlands

## Abstract

Neuronal activity in early visual cortex depends on attention shifts but the contribution to working memory has remained unclear. Here, we examine neuronal activity in the different layers of the primary visual cortex (V1) in an attention-demanding and a working memory task. A current-source density analysis reveales top-down inputs in the superficial layers and layer 5, and an increase in neuronal firing rates most pronounced in the superficial and deep layers and weaker in input layer 4. This increased activity is strongest in the attention task but it is also highly reliable during working memory delays. A visual mask erases the V1 memory activity, but it reappeares at a later point in time. These results provide new insights in the laminar circuits involved in the top-down modulation of activity in early visual cortex in the presence and absence of visual stimuli.

## Abstract (web summary)

The effect of working memory on activity in primary visual cortex (V1) is not well understood. Here the authors report a clear influence of both working memory and attention on spiking activity in the superficial and deep layers of V1 with a weaker influence on input layer 4.

Selective attention and working memory are essential in daily life. While attention serves to select relevant stimuli, working memory retains the information when the stimulus has disappeared. At the neuronal level, selective attention is thought to cause a top-down modulation of neuronal activity in sensory areas^1^,^2^,^3^. The cortical mechanisms for memory are less well understood. It is generally believed that visual memories consist of a number of phases that differ in their stability. The first phase is iconic memory, a high capacity store that lasts about 100 ms and resembles a snapshot of what was just seen^4^,^5^. Iconic memory traces are fragile and are overwritten when new information is presented. During the decay of iconic memory, a subset of visual items can be transferred into visual working memory^6^,^7^, which is a more robust memory store that can last several seconds but has a small capacity^8^. At the neuronal level, iconic memory is thought to correspond to the decaying activity that follows the response elicited by the stimulus in low-level areas of the visual cortex. Neuronal activity underlying the more stable working memories occurs in higher areas of the visual, parietal and frontal cortex where neurons exhibit persistent firing, even after the stimulus has disappeared^9^,^10^,^11^. The role of low-level areas in the maintenance of relevant visual information is under debate^12^. On the one hand, a recent study^13^ demonstrated that persistent firing during working memory for the movement direction of a briefly presented stimulus is virtually absent from the middle temporal (MT) area, a lower-level motion-sensitive area, but that it is strong in the next higher medial superior temporal (MST) area and in the frontal cortex. This finding suggests that persistent firing is a unique property of higher cortical areas. On the other hand, studies in human observers demonstrated that memory traces of low-level stimulus attributes may persist for seconds^14^, and fMRI studies revealed that is it possible to decode the orientation or colour of a stimulus in working memory from activity in primary visual cortex (V1), in accordance with ‘sensory recruitment' theories postulating that vivid memories require feedback from higher cortical areas to reinstate activity patterns in sensory cortices^15^,^16^,^17^,^18^. Indeed, a recent fMRI study demonstrated that contextual influences in the absence of visual input are strongest in the superficial layers of V1, suggesting a role for feedback from higher visual areas^19^. However, it not known whether the fMRI signals elicited by working memories reflect only subthreshold synaptic events or whether neurons also increase their spiking activity^20^,^21^,^22^. One electrophysiological study in monkeys demonstrated that working memory influences firing rates in V1 (ref. 23), but used a stimulus with texture elements that drove the neurons during the delay period. It is therefore unknown if V1 neurons exhibit persistent activity when there is no stimulus in the receptive field (RF), and how feedback from higher visual areas is involved in this process.

To address these questions, we here trained monkeys to perform a curve-tracing task, which requires an analysis of the location and orientation of multiple contour elements that are represented at a high resolution in the lower areas of the visual cortex. We illustrated an example of curve-tracing stimuli in Fig. 1a–d. The monkey's task was to determine the green circle that connected to the fixation point by a target curve, and to make an eye movement to this circle after a delay. Previous studies used a version of this task where the stimulus remained in view and demonstrated that the feedforward response is followed by a phase where horizontal connections and feedback connections modulate V1 activity^24^,^25^,^26^. In this later phase, enhanced neuronal activity spreads gradually along the V1 representation of the target curve, starting at the fixation point until the entire curve has been labelled with enhanced neuronal activity^24^,^27^, a process that corresponds to the spread of object-based attention at a psychological level of description^28^,^29^. During this later phase of neuronal activity, image elements far from the neurons' RFs influence V1 activity, and information about these image elements can only reach the neurons through horizontal connections within V1 and through feedback from higher visual areas (reviewed by ref. 25). The speed at which the enhanced neuronal activity spreads over the target curve is comparable to the speed with which human observers trace the curve^27^. Furthermore, it was recently shown that neuronal firing rates in the human visual cortex elicited by target curves are higher than those elicited by distractors, just as is the case in monkey visual cortex^30^. Thus, the curve-tracing task is useful to study the top-down influences related to perceptual organization and object-based attention in the visual cortex^25^. Human observers can continue tracing for a few hundred milliseconds if the stimulus is presented only briefly^31^. This capacity to trace a mental image of a curve implies that the observers must have access to the curves' precise shapes even though the stimulus is no longer in view. We therefore hypothesized that early visual areas might also contribute to curve tracing after the stimulus has disappeared.

We trained monkeys in a curve-tracing task where the stimulus either remained on the screen for the complete duration of the trial, or was only briefly presented and used a laminar electrode to record the activity of neurons in the different layers of V1. This approach enabled us to address a number of questions. First, we wanted to test if monkeys can trace briefly presented curves. If so, do V1 neurons exhibit persistent activity that depends on the relevance of previously presented contour elements? Second, we aimed to compare the putative V1 memory signal to the attentional response modulation when the curves remain visible. Third, we wanted to measure the influence of a visual mask on the activity in area V1 because masking interferes with the more fragile iconic memories whereas working memories resist masking^32^. Fourth and finally, we wished to compare the different V1 layers, which might contribute differentially to attention and working memory^33^,^34^.

We find that monkeys can trace curves that are no longer in view and that this mental tracing process causes persistent spiking activity in V1, implying a form of working memory. The V1 memory trace is briefly abolished by a mask but it is subsequently reinstated, implying that it provides a neural correlate of working memory and not of iconic memory. We note, however, that our task does not dissociate working memory within V1 from a top-down, spatially selective influence on V1 by working memories stored elsewhere. For simplicity, we will refer to the neuronal correlate of selecting a curve that has disappeared as ‘working memory' and reserve the term ‘attention' for situations where the stimulus remained in view. Furthermore, we observe that the modulation of spiking activity by attention and working memory is strongest in the superficial and deep layers of V1 and weaker in input layer 4. A current-source density analysis indicates that both cognitive functions increase the magnitude of synaptic input into the superficial layers and layer 5, thus providing a canonical signature of top-down influences from higher visual cortical areas back to V1.

## Results

### Layer-specific activity in V1 during the curve-tracing task

**Figure 1:**
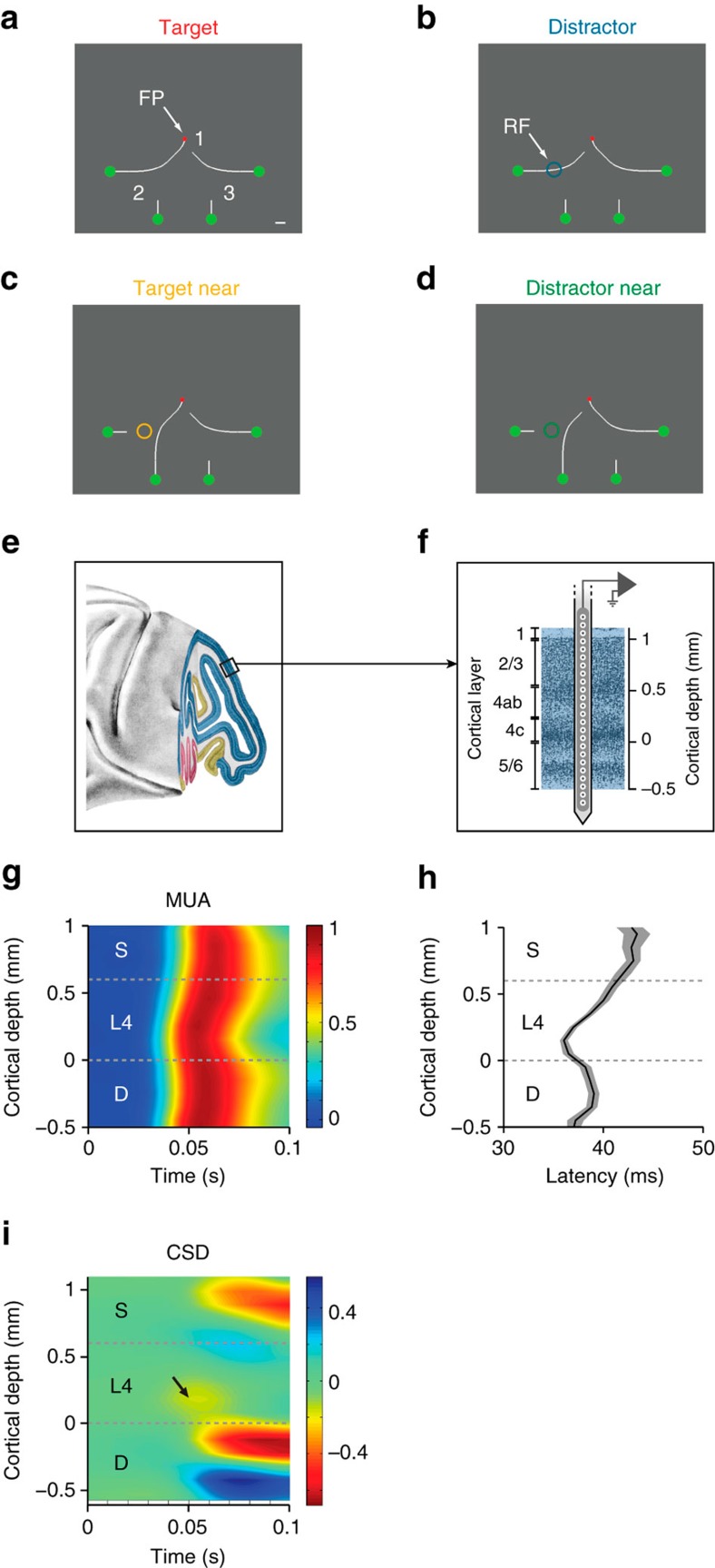
Laminar recordings and visual stimuli. (**a**–**d**) The monkey had to mentally trace the target curve that was connected to the fixation point (FP), and to make an eye movement to the green circle at the end of this curve. We placed the target curve (**a**) or a distractor curve in the RF (**b**). In other conditions the target (**c**) or distractor curve (**d**) was next to the RF. Circles, RFs. Bar in **a**, 1°. (**e**) Lateral view of the macaque brain. V1 is the blue region. (**f**) Laminar recording with the multi-site linear electrode (Plexon Inc. U-probe). (**g**) The average MUA response evoked by the onset of the target curve across the layers. Note the slight differences in the timing of the onset of the MUA response between layers. (**h**) Visual latencies in the different layers, averaged across all penetrations, shaded areas indicate s.e.m. (*n*=38 penetrations). The earliest neuronal activity occurred in layers 4C and 6. (**i**) Average CSD evoked by the appearance of the target curve. Warm colours indicate current sinks, cooler colours current sources. The appearance of the curve causes an early sink in layer 4C (arrow). (**e**,**f**) Adapted from ref. 39.

**Figure 2:**
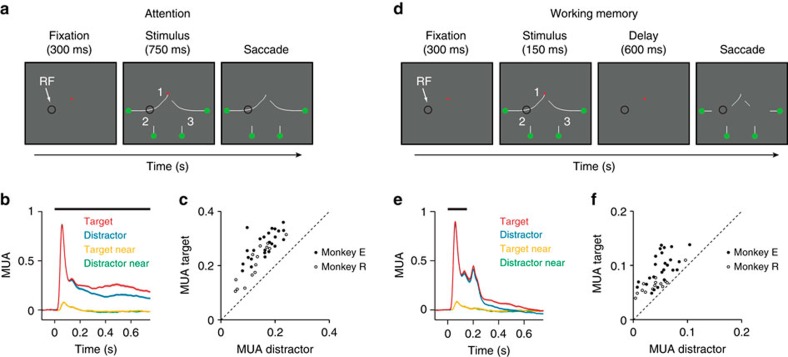
The influence of attention and working memory on spiking activity. (**a**,**d**) After a 300 ms fixation period, four curves appeared on the screen. The monkeys had to make a saccade to a green circle at the end of the target curve connected to the fixation point. In the attention task (**a**) the stimulus remained on the screen whereas the stimulus disappeared after 150 ms in the memory task (**d**). After an additional delay (600 ms), part of the stimulus reappeared, but the segments at locations 2 and 3 did not, so that the animal had to remember the configuration of the target curve. Black circle, typical RF location. (**b**,**e**) Neuronal activity averaged across all V1 recording sites in the attention (**b**) and the memory task (**e**). The RF fell on the target (red trace), distractor curve (blue), or next to the target (yellow) or distractor curve (green). Shaded areas show s.e.m. (*n*=37 penetrations; when they are difficult to see the s.e.m. is small). (**c**,**f**) Data of the individual penetrations in monkeys E (black data points) and R (white) in the attention (**c**) and working memory task (**f**). Abscissa, MUA elicited by the distractor curve (200–750 ms after stimulus onset). Ordinate, MUA elicited by the target curve.

We recorded multi-unit activity (MUA) and local field potentials (LFPs) in the different layers of monkey V1 using a high-density depth probe with a spacing of 100 μm between electrodes (Fig. 1e,f). We used a version of a curve-tracing task^24^ that allowed a direct comparison between selective attention for stimuli that remained on the screen and working memory for stimuli that were only presented briefly. The monkeys directed their gaze to a red fixation point and we then presented four curves with green circles at their ends (Fig. 1a–d). After a delay the fixation point disappeared, cueing the monkey to make an eye movement to the green circle that was connected to the fixation point (for example the left circle in Fig. 1a). The initial contour segment at location 1 connected the fixation point to the left or the right main branch, and the contour segments at locations 2 and 3 established a connection between the main branches and one of two sub-branches and hence the saccade target. At each of the three locations the contour elements could be in one of two configurations, so that there were a total number of eight stimuli (Fig. 1a–d shows the variations at locations 1 and 2, Supplementary Fig. 1 shows all stimuli). We adapted the stimulus so that the neurons' RF fell on one of the possible segments at location 2, and the configuration at location 1 determined whether this segment belonged to the target curve (Fig. 1a) or a distractor curve (Fig. 1b), while RF stimulation was constant. For the two other stimuli the target (Fig. 1c) or distractor curve was adjacent to the RF (Fig. 1d). In the attention task, the stimulus was always visible and the fixation point disappeared after 750 ms, cueing the monkey to make a saccade (Fig. 2a). In the memory task, the stimulus disappeared after 150 ms and only part of the stimulus reappeared when the monkey was cued to respond (Fig. 2d). We held the orientation of the contour element in the neurons' RF constant to obtain a sufficient number of trials per condition, and we therefore did not determine orientation tuning.

In the attention task (performed in blocks of ∼100 trials per condition), the monkeys' accuracy was high (94% for monkey E and 97% for monkey R). The appearance of a contour segment in the RF elicited a feedforward MUA response in the different V1 layers, starting in layers 4C and 6 and then spreading into the superficial and deep layers (Fig. 1g,h). We also computed the current-source density (CSD) profile to determine the putative location of synaptic inputs. The small contour element in the RF elicited a relatively weak initial current sink in layer 4C (arrow in Fig. 1i), which was followed by current sinks and sources in the superficial and deep layers^34^,^35^. This laminar pattern is consistent with the projection from the lateral geniculate nucleus (LGN) to layer 4C (ref. 36) which, in turn, targets the superficial and deep layers^37^, although the later CSD profile presumably also includes contributions from horizontal and feedback connections^34^. There are also weaker projections from the LGN to layer 6 and the superficial layers^36^,^37^, but these inputs are not visible in the CSD because the current sink in layer 4 is stronger.

The crucial segment at location 1 was far from the V1 RF (Fig. 1a,b) so that the feedforward input from the LGN for the stimuli with the target and distractor curve in the RF was constant. Accordingly, the initial MUA response did not distinguish between the target and distractor curve (Fig. 2b). However, after a delay the representation of the target curve was enhanced over the representation of the distractor (200–750 ms after stimulus onset, Wilcoxon signed-rank test, monkey E: *n*=24 penetrations, *P*<0.001; monkey R: *n*=13 penetrations, *P*<0.001), in line with previous studies demonstrating that the enhanced V1 response is a neuronal correlate of object-based attention spreading across all contour elements of the target curve^24^,^25^,^27^. The reliability of the attentional modulation in V1 was high as it occurred in every penetration (Fig. 2c). A linear decoder based on spiking activity across the layers could discriminate the target from the distractor in single trials with an accuracy 78% for monkey E and 66% for monkey R (cross-validation accuracy). A target or distractor curve that fell next to the RF elicited only a weak, transient and delayed response (Fig. 2b)^38^ that did not discriminate between target and distractor (Wilcoxon signed-rank test, *P*>0.1 for both monkeys). Thus, attention only influenced neurons that were well activated by a contour element in their RF.

In alternating blocks of ∼100 trials we examined whether the monkeys were able to trace curves that were presented for 150 ms, followed by a delay of 600 ms with only the fixation point left on the screen. At the end of the trial, the stimulus reappeared, but without the line elements at locations 2 and 3, so that these line elements had to be remembered (Fig. 2d). The monkeys' performance was comparable to that in the regular curve-tracing task (90% for monkey E and 97% for monkey R; Wilcoxon signed-rank test, *P>*0.1 for both monkeys), suggesting that they could trace the image of a curve that had been presented only briefly, just like human observers^31^. In this version of the task, the MUA exhibited an off-response and the activity then gradually decreased to baseline (Fig. 2e), a decrease in activity that presumably corresponds to the decay of iconic memory. Strikingly, the response elicited at the location where the target curve had appeared remained stronger than at the distractor location for the full duration of the trial (Fig. 2e) (Wilcoxon signed-rank test in a window 200–750 ms after stimulus onset, monkey E: *n*=24 penetrations, *P*<0.001; monkey R: *n*=13 penetrations, *P*<0.001). Although this modulation of persistent activity was weaker than the attentional modulation in the presence of the stimulus (Wilcoxon rank-sum test, monkey E: *n*=24 penetrations, *P*<0.001; monkey R: *n*=13 penetrations, *P*<0.01), it was present in all penetrations in both monkeys (Fig. 2f). Furthermore, the classification accuracy was significant for target versus distractor trials (mean accuracy of 0.77 for monkey E and 0.63 for monkey R; permutation test, *P*<0.01 for both monkeys). No modulation of V1 activity occurred when the target or distractor curve was adjacent to the RF (*P*>0.05 for both monkeys), indicating that the memory signal was either spatially highly specific or depended on the preceding brief stimulus in the neurons' RF. A previous study demonstrated that working memory also modulates the power spectrum of the LFP in early visual cortex^13^. We therefore also examined the LFP and observed that the target curve elicited more power in the gamma range (30–90 Hz) and less power in the alpha range (5–15 Hz) than the distractor curve, in both the attention and the working memory tasks (Supplementary Fig. 2; *P*<0.001 for both monkeys and both comparisons). These results support previous findings that increases in spiking activity are usually associated with more gamma power and less power at lower frequencies in the LFP^39^.

**Figure 3:**
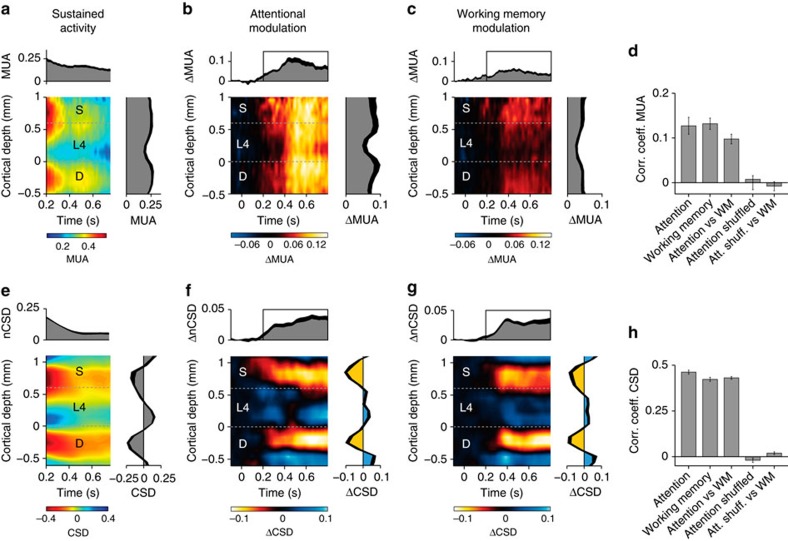
The laminar profile of selective attention and working memory. (**a**) Laminar pattern of spiking activity in the epoch after the peak response. (**b**,**c**) The attentional modulation (**b**) and working memory effect (**c**) on spiking activity across the depth of the cortex, which is the difference in activity elicited by the (memory of) the target and the distractor curve (red minus blue traces in Fig. 2b,e). Graphs on the right and above show the averages across layers and across the modulation period (black rectangles in the upper graphs; 200–750 ms after stimulus onset), respectively. The thick black traces represent s.e.m. (**d**) Consistency of the laminar MUA profile as assessed with an analysis of correlations between penetrations and between the attention and working memory conditions. Also shown are controls when the data are shuffled. Error bars indicate s.e.m. (**e**) Average CSD during the episode when the peak response has subsided. The average laminar CSD profile is plotted on the right. The upper panel shows the inner product of this profile with the CSD at the different time points (nCSD). (**f**,**g**) Average difference in CSD between target and distractor curve. (**h**) Analysis of the consistency of the laminar CSD profile across penetrations by computing pair-wise correlations. *n*=37 penetrations in all plots.

We next examined the activity profile in the presence and absence of the stimulus across the depth of the cortex. Figure 3a shows the sustained spiking activity (200–750 ms after stimulus onset; *n*=37 penetrations) elicited by a contour element of the distractor curve (blue in Fig. 2b) across the layers. For statistical analysis we normalized activity at every electrode to the peak response and we assigned electrodes to three compartments, superficial layers, layer 4 and the deep layers (see ‘Experimental Procedures'). The sustained activity level differed significantly between laminar compartments (one-way repeated measures analysis of variance (ANOVA), monkey E: *F*(2,46)=3.7, *P*<0.05; monkey R: *F*(2,24)=17.1, *P*<0.001). It was stronger in the superficial and deep layers than in layer 4 (post-hoc Wilcoxon signed-rank test, superficial/deep layers versus layer 4, both *P*s<0.005 for both comparisons and both monkeys). There were also differences between layers in the strength of the attentional modulation (Fig. 3b; one-way repeated measures ANOVA, monkey E: *F*(2,46)=9.4, *P*=0.001; monkey R: *F*(2,24)=6.63, *P*=0.005), which was strongest in the superficial and deep layers and weaker in layer 4 (superficial/deep layers versus layer 4, *P*s<0.01 for both monkeys and comparisons), in accordance with the anatomy of feedback connections to V1, which tend to avoid layer 4 (refs 40, 41). The laminar profile of attentional modulation was consistent across penetrations (Fig. 3b,d) (test of significant correlation of laminar profiles across penetrations; Wilcoxon signed-rank test, monkey E: *P*<0.001; monkey R: *P*<0.01; unlike a shuffle control, *P*>0.4 for both monkeys).

When the stimulus disappeared, the difference between activity elicited by the target and distractor was weaker than when it remained on the screen, but the laminar profile was similar (Fig. 3c), with a significant difference between the layers (one-way repeated measures ANOVA, monkey E: *F*(2,46)=12.7, *P*<0.001; monkey R: *F*(2,24)=4.7, *P*=0.025). The extra activity elicited by the target curve was more pronounced in the superficial and deep layers than in layer 4 (monkey E: superficial/deep layers versus layer 4, both *P*s<0.001; monkey R: superficial/deep layers versus layer 4, both *P*s<0.01). This laminar profile of MUA modulation was similar between tasks (significantly correlated in both monkeys: *P*s<0.01) (Fig. 3d), suggesting a common feedback source for selective attention and working memory.

**Figure 4:**
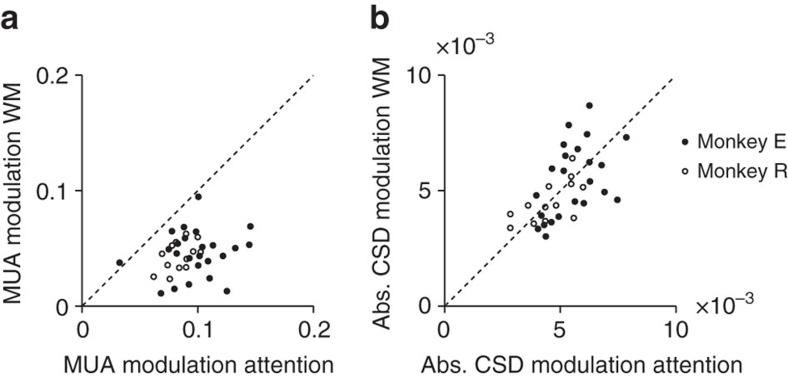
Comparison of attentional and working memory modulation across penetrations. Modulation of the MUA (**a**) and the absolute value of the modulation of the CSD (**b**) averaged across layers, for attention (ordinate) and working memory (abscissa) from 200 to 750 ms after stimulus onset. The modulation of the MUA was stronger in the attention task than during the delay epoch of the working memory task (monkey E: *n*=24 penetrations, *P*<0.001; monkey R: *n*=13 penetrations, *P*<0.01), whereas the modulation of the CSD did not differ significantly between tasks (both *Ps*>0.7).

We calculated the CSD to investigate the synaptic sources underlying the extra MUA caused by attention and working memory. If the distractor curve remained visible, it evoked two sinks, one in the deep layers and the other one straddling the superficial layers and upper layer 4 (Fig. 3e). When we subtracted the distractor CSD from the target CSD to isolate attentional modulation, we identified two sinks that were more confined. One was in the superficial layers and the other one in layer 5 (Fig. 3f), which are the layers that receive feedback connections from higher visual areas^40^,^41^. This CSD pattern was highly significant and consistent across recordings (Fig. 3h; Supplementary Fig. 3a for an analysis based on cluster statistics). The CSD associated with the modulation of persistent activity in the memory task exhibited a surprisingly similar laminar profile compared with the attention task (200–750 ms after stimulus onset; *P*<0.001 for both monkeys) (Fig. 3h), with sinks in layers 1–3 and 5 (Fig. 3g; Supplementary Fig. 3b). Thus, both tasks gave rise to similar laminar patterns of spiking activity and synaptic input, in accordance with the anatomy of feedback connections to V1. The amplitude of the CSD modulation was also similar in the presence and absence of the stimulus (Fig. 4b) (absolute value of the modulation of the CSD, averaged across layers; Wilcoxon signed-rank test, *P*>0.7 for both monkeys), whereas the MUA modulation was stronger with visible curves (Fig. 4a) (monkey E: *n*=24 penetrations, *P*<0.001; monkey R: *n*=13 penetrations, *P*<0.01). This result implies that the presence of a curve in the RF amplified the top-down influence on firing rates, but it should not distract from our finding that a briefly presented curve causes a highly reliable modulation of persistent activity in V1 during the ensuing memory epoch.

### Dissociating the memory trace from the saccade plan

**Figure 5:**
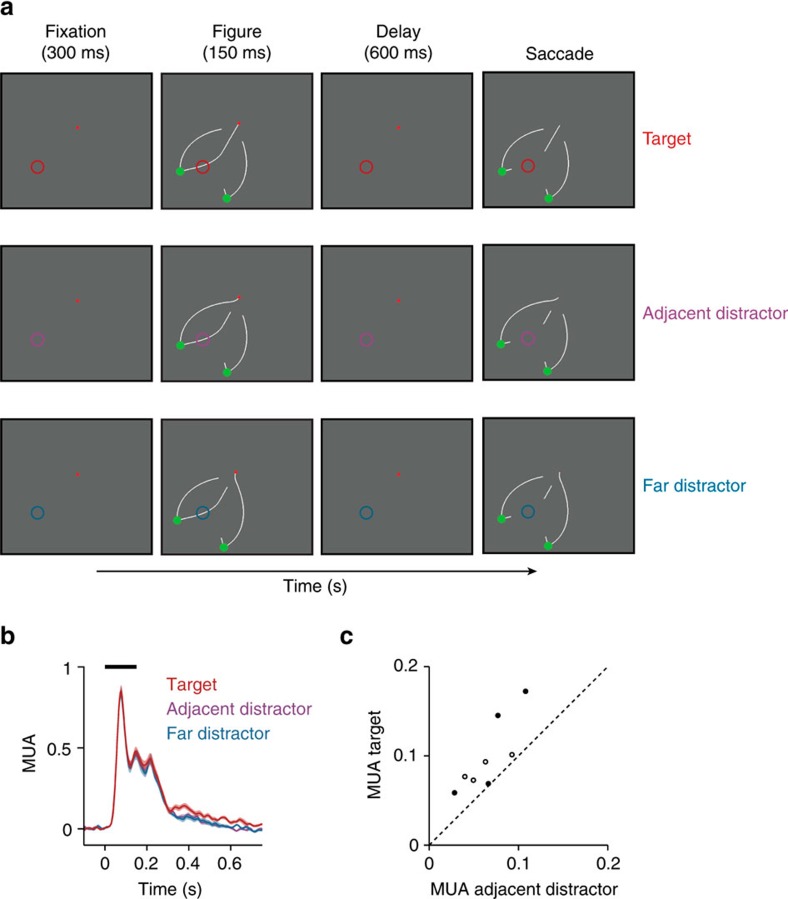
Eye movement control. (**a**) Stimulus with three curves and two saccade targets. To dissociate memory of the target curve from the saccadic eye movement plan, we compared activity elicited by the target curve (upper row) by an adjacent distractor curve (middle row) associated with the same saccade and by a far distractor curve associated with a different saccade (lower row). (**b**) The MUA response averaged across all V1 recording sites elicited by the target (red trace), the adjacent distractor (magenta trace), and the far distractor curve (blue trace). Shaded areas show s.e.m. (*n*=8 penetrations). The response depended on the memory of the target curve and not on the saccade plan. (**c**) MUA elicited by the memory of the target curve and the adjacent distractor in a window from 200 to 750 ms after stimulus onset (filled circles for monkey E, open circles for monkey R).

Previous studies have demonstrated that the entire target curve is labelled with enhanced neuronal activity during curve-tracing and that the V1 response modulation can be dissociated from the saccade plan^24^,^42^. It is unknown, however, if the modulation of persistent activity in the task in which the stimulus disappears can also be dissociated from the trajectory of the impending eye movement. Specifically, if the neurons' RF fell on the previous location of the target curve, then it was also closer to the path of the planned saccade (Fig. 1). We therefore designed a control task where the RF could fall either on the target or on a nearby distractor curve with an identical saccade target (Fig. 5a, see Supplementary Fig. 4 for all stimuli). The RF could also fall on a distractor curve that was farther away. The average MUA response elicited by the memory of the target curve was stronger than that elicited by the near distractor (*n*=4 penetrations in each monkey, both *P*s<0.05) and the far distractor (both *P*s<0.05) (Fig. 5b,c). Thus, persistent activity was strongest for task-relevant parts of the memory representation and it was not due to eye movement preparation.

### The influence of masking on persistent activity in V1

**Figure 6:**
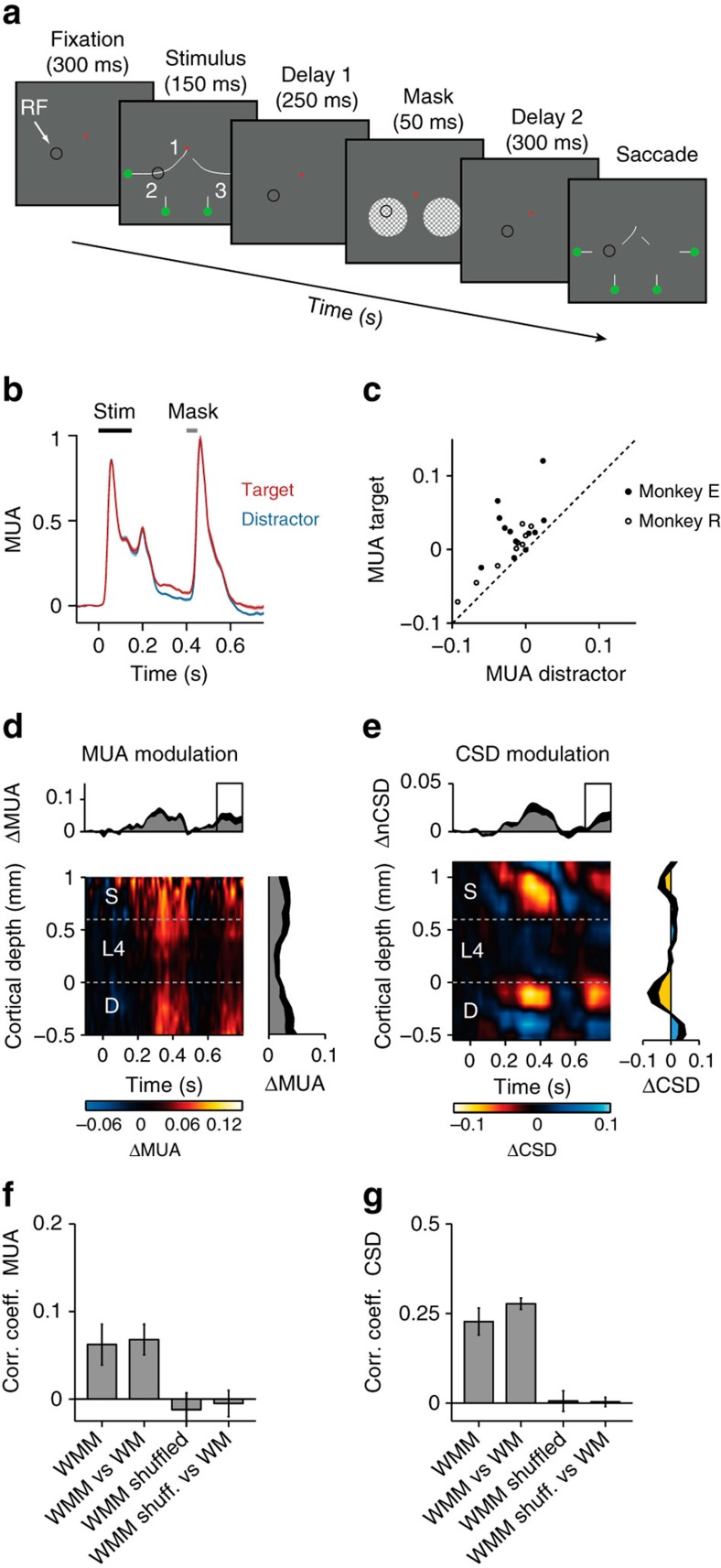
The influence of a mask. (**a**) The stimulus was shown for 150 ms, and 250 ms later a mask appeared (50 ms), followed by another delay of 300 ms. (**b**) Neuronal activity averaged across all V1 recording sites in two monkeys evoked by the target (red trace) and the distractor curve (blue) (monkey E: *n*=12 penetrations, monkey R: *n*=9). Shaded areas show s.e.m., if it is difficult to see, the s.e.m. is small). (**c**) MUA evoked by the target (ordinate) and distractor curve (abscissa) during delay 2 (650–750 ms after stimulus onset). (**d**) Difference in MUA elicited by the target and distractor curve across cortical depth. Upper panel, average across layers. Right panel, average in window from 650 to 750 ms after stimulus onset (black square in upper panel). (**e**) CSD difference between target and distractor curve. Upper panel, time course (inner product with the average laminar profile from 650 to 750 ms). Right panel, average from 650 to 750 ms. Black lines, s.e.m. (**f**,**g**) Correlation coefficients of the MUA (**f**) and CSD (**g**) across penetrations from 650 to 750 ms. WMM, consistency across penetrations of working memory modulation after the mask. WMM versus WM, correlation coefficient between WM modulation after the mask and when there was no mask. Error bars, s.e.m.

Is the modulation of persistent activity a spatial working memory trace or does it represent an influence of attention on iconic memory? Iconic memories are sensitive to masking, while information becomes resistant against masks once it has been transferred into working memory^4^,^8^,^43^. In our next experiment we therefore tested the influence of masks on persistent firing in V1. We showed two full contrast checkerboard stimuli at locations 2 and 3, 400 ms after the onset of the stimulus (250 ms after the offset). One of these masks covered the neurons' RFs (Fig. 6a). The monkeys' accuracy in this task was lower than in the memory task without a mask (84% for monkey E, *P*<0.05; and 95% for monkey R, a trend in the same direction, *P*=0.1, Wilcoxon rank-sum test). The mask elicited a strong MUA response that abolished the curve-tracing modulation (non-significant modulation in a window from 450 to 550 ms after stimulus onset, *P>*0.3 for both monkeys, monkey E: *n*=12 penetrations, monkey R: *n*=9). Notably, the modulation returned ∼200 ms after mask offset (Fig. 6b; *P*s<0.01 for both monkeys), an effect that occurred for nearly all penetrations in both monkeys (Fig. 6c). The magnitude of the MUA modulation in this epoch was similar to that when no mask had been shown (650–750 ms after stimulus onset, *P*>0.4 for both monkeys) and the profile across the layers was similar (Fig. 6d,f). The mask also disrupted the characteristic CSD profile with sinks in the superficial layers and layer 5 (Fig. 6e), associated with the selection of the target curve in memory, but the CSD pattern reappeared (Fig. 6e; 650–750 ms after stimulus onset, absolute value of the modulation of the CSD across layers; both monkeys *P*<0.01). During this phase, the CSD profile was similar to that in the task without a mask (Fig. 6g). Thus during the mask, V1 spiking activity does not store the memory of the stimulus but the modulation of spiking activity is restored later, presumably due to feedback from higher areas. The reappearance of the V1 modulation suggests that these putative higher areas enable a more stable form of memory, and it excludes the possibility that the modulation of the V1 response depends on iconic memory.

### Working memory for curves has a limited capacity

**Figure 7:**
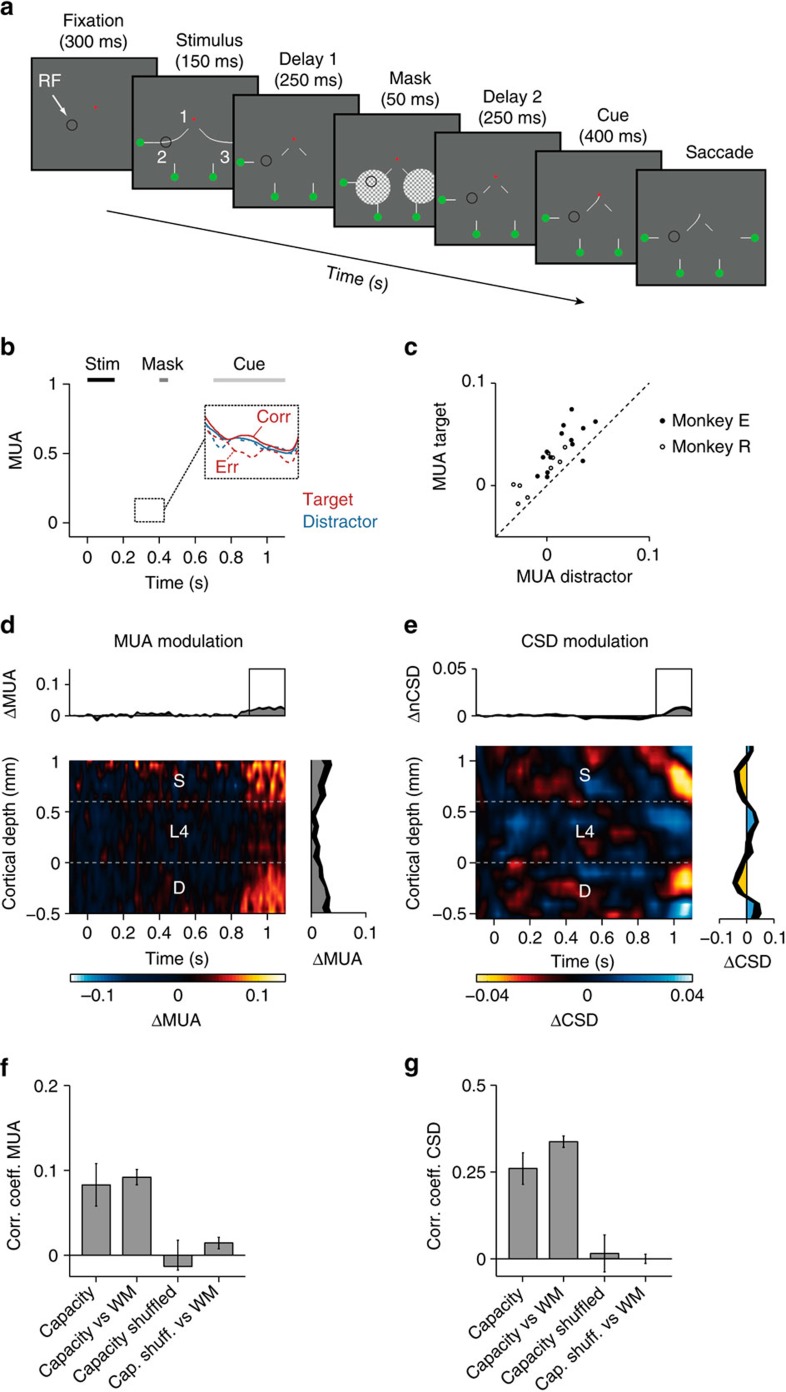
Capacity limitations in working memory. (**a**) The contours in or near the RF disappeared after 150 ms and a mask (50 ms) appeared after 250 ms, followed by a second delay of 250 ms. The connection to the fixation point was shown after the second delay, cueing one of the curves as target. (**b**) Average MUA response evoked by the target (red trace) and the distractor curve (blue). Shaded areas show s.e.m. (*n*=23 penetrations). Inset, activity elicited by the future target and distractor curve on correct (continuous curves) and erroneous trials (dashed) before the central cue identified the target. Note that reduced memory activity for the target curve is predictive of an error. (**c**) MUA elicited by target (ordinate) and distractor (abscissa) from 850 to 1100, ms after stimulus onset. (**d**,**e**) Difference in MUA (**d**) and CSD (**e**) elicited by target and distractor curve. (**f**,**g**) The left bar represents the average correlation coefficients of the laminar pattern of the MUA (**f**) and CSD (**g**) between penetrations, from 850 to 1,100 ms. The second bar (capacity versus WM) compares the laminar profile to that in the memory task of Fig. 2d. Error bars show s.e.m.

We next investigated whether the monkey could maintain the configuration of two curves in memory by revealing the cue at location 1 only after the crucial segments at locations 2 and 3 had disappeared and been masked (Fig. 7a). To achieve perfect performance, the monkeys now had to memorize both segments at locations 2 and 3. The 'skeleton' of the stimulus remained in view, to aid the monkeys with a task that was relatively difficult for them. The accuracy was lower than in the task of Fig. 2d (74% for monkey E and 81% for monkey R; both *P*s<0.001) and close to 75%, as if the monkeys could hold only one of the masked segments in memory (for example, good memory for the configuration at location 2 or 3 with an accuracy of 100% if the target curve was on that side and poor memory with 50% accuracy if the target curve was on the other side). As expected, the MUA response elicited by the curves that would later become target or distractor were indistinguishable during the first delay (Fig. 7b), but ∼200 ms after the appearance of the cue at location 1, the representation of the previous target curve increased (900–1,150 ms after stimulus onset, *P*s<0.005 in both monkeys). This late modulation of spiking activity occurred in nearly all penetrations in both monkeys (Fig. 7c) and exhibited its characteristic laminar pattern (Fig. 7d,f). This was also true for the laminar CSD pattern (Fig. 7e,g), which was significant (900–1,150 ms after stimulus onset, absolute value of CSD modulation across layers; both *P*s<0.01). This result suggests that the cue near the fixation point initiated a feedback signal from higher areas to V1, which enhanced the representation of the memory of the relevant curve in V1.

The monkeys' accuracy suggested that they could memorize, on average, only one of the two masked configurations at locations 2 and 3. We therefore hypothesized that V1 activity during the first delay might predict which segment was remembered and compared the neuronal activity between correct and erroneous trials. In this analysis we only included error trials where the monkeys chose the correct main branch at location 1, but made a mistake at location 2 or 3. On the side of the stimulus that would later become the target, V1 delay activity was stronger on correct than on erroneous trials (Fig. 7b, inset) (*n*=14.9 in monkeys E and R, respectively, both *P*s<0.05). If the future distractor was in the RF, however, delay activity was slightly, but not significantly weaker on correct trials (*P*=0.2 for monkey E and *P*=0.1 for monkey R). Thus, V1 activity during the pre-mask delay predicted the quality of the memory for the contour element in the RF.

## Discussion

The present study is the first to investigate the influence of working memory on the activity of neurons in the different layers of area V1. We capitalized on the ability of observers to trace curves that are presented only briefly, as if they inspect a mental image of a previously presented stimulus at a high spatial resolution^31^. We compared the mechanisms of this mental tracing process to the mechanisms for curve tracing with the stimulus in view, thereby directly comparing the neuronal correlates of spatial attention and working memory. We found that monkeys are also able to trace curves that are only briefly shown to them, just like human observers. The relevance of contour elements modulated the spiking activity of V1 neurons, during a phase in which they were no longer in view. The enhanced V1 activity was restricted to the precise location where the curves had been presented, in line with the hypothesis that the monkeys traced a mental image of the target curve. These results support studies that demonstrated short-term memory for location^23^ and figure-ground organization^44^ in early visual cortex while driving cells with a texture or an edge, and also go beyond by demonstrating working memory signals in the complete absence of a visual stimulus.

The direct comparison of the influence of selective attention and working memory on neuronal activity in the different layers of cortex revealed an almost identical CSD profile, suggesting that the mechanisms for selective attention overlap with those for working memory^45^,^46^,^47^. Attention and working memory caused current sinks in the superficial layers and layer 5 of similar magnitude, yet the modulation of MUA was highest with a stimulus in the neurons' RF, indicating that the feedforward drive of a stimulus enhances the impact of feedback on firing rates.

Theories about visual memory for briefly presented stimuli distinguish between an early iconic memory store with a rapid decay and a later working memory store that lasts longer^4^,^43^. A distinguishing feature of iconic memory, which differentiates it from working memory, is its sensitivity to masking. Masks erase iconic memories but they usually do not interfere with items that have been transferred into working memory. The transfer from iconic memory to working memory has to be selective because the capacity of working memory is much smaller than that of iconic memory^4^,^43^,^48^. Although the delays tested by us were slightly shorter than in previous studies of working memory, our results imply that the V1 response modulation is a neuronal correlate of working memory, for a number of reasons. First, the delays tested by us (up to 950 ms, Fig. 7a) exceed the persistence of iconic memory^5^. Second, the memory recovered after a mask. Third, we found that the monkeys' capacity to memorize contour configurations was limited and that V1 activity predicted the quality of the memory for the curve in the RF. Thus, the late modulation of V1 activity provides a neuronal correlate of working memory. At the same time, it is conceivable that the early visually driven but quickly decaying V1 response provides a neuronal correlate of iconic memory, a conjecture that could be tested in future work.

Many previous studies in monkeys performing working memory tasks focused on higher cortical areas where the persistent firing for items in working memory can be strong^9^,^10^,^11^. It has remained less clear if working memory also relies on persistent activity in early visual cortical areas^12^. On the one hand, fMRI studies in humans reveal signatures of the items in working memory in higher but also in lower visual areas^7^,^47^,^49^. Indeed, it is possible to decode the stimulus in memory from early visual areas by analysing the pattern of the fMRI signal across multiple voxels^15^,^50^, even when the visually driven fMRI response has decayed back to baseline^51^,^52^. One the other hand, it was unknown whether these fMRI signals reflect an influence of memory on spiking activity or on subthreshold synaptic activity^20^,^21^,^22^. In accordance with such a subthreshold influence, a recent study on the influence of working memory on neuronal firing rates reported that persistent firing was virtually absent from motion sensitive area MT, but pronounced in the higher visual area MST and prefrontal cortex^13^. Nevertheless, the present results reveal that tracing a curve in memory modulates V1 spiking activity, during a phase where the overall V1 firing rate is close to spontaneous activity levels. It is of interest that masks briefly eliminated this memory trace, but that it reappeared after the mask. Although these results do not rule out local storage of information within V1 by processes other than spiking activity, such as rapid synaptic potentiation^53^, it seems more likely that the target curve was stored in higher cortical areas, which then fed back to restore the pattern of response modulation in V1. There are several candidate structures that could have acted as a source of these top-down signals. First, the inferotemporal cortex might store the shape of the target curve during the memory delay^54^. Second, neurons in the parietal cortex have been shown to code for the configuration of image elements that are part of a perceptual group^55^. Third, memory representations in the frontal cortex are relatively uninfluenced by intervening stimuli, that is, they resist masking^11^. Although the direct feedback connections of some of these higher cortical regions areas to area V1 are relatively weak^56^, these top-down influences can also be mediated indirectly, via areas such as V4 and V2.

We do not know why we observed such a robust influence of memory on V1 activity, which was seen in virtually all of our recordings, where previous studies failed to find such an effect. One possibility is that tracing a curve in memory necessitates the access to a high-resolution representation of the location and orientation of contour elements, which can be made available by V1 after sufficient training. Compared with other tasks, the attentional selection of contour elements during the tracing of visible curves also causes a relatively strong modulation of V1 activity compared with some other tasks that demand attention shifts^24^,^57^. Another difference is that we investigated spatial working memory for a relevant contour, whereas previous studies focused on memory features other than space, like the direction of motion. It is therefore also possible to account for our results with a sustained spatial attention signal that is directed to the locations that were previously occupied by a relevant curve, although one previous study reported that attention does not influence ongoing V1 activity if there is no stimulus in the neurons' RF^57^. It is generally difficult, if not impossible, to dissociate working memory for spatial locations from sustained attention to these locations. Indeed, the presence of a sustained spatial attention signal in the absence of a stimulus implies that the to-be-attended locations are stored in memory. Interestingly, this working memory trace specifically encoded the location of the target curve with a high degree of spatial specificity (Figs 2e and 5), which suggests that it was contingent on the preceding visual response^58^ and implies that it cannot be explained by a spatially diffuse top-down signal.

Although models of the visual system often emphasize the importance of feedforward connections^59^,^60^,^61^, early visual areas receive at least as much feedback as feedforward input^56^. Area V1 is an extreme case, because only ∼1% of the external input connections come from the LGN and ∼95% from higher visual areas^56^. Feedforward and feedback connections target different V1 layers^36^,^40^,^41^, which allowed us to probe these two processing streams separately. The visually driven spiking response started in input layer 4 (refs 62, 63) and then quickly spread to the superficial and deep layers. In accordance with previous work, the initial visual response coincided with a sink in layer 4, the target of feedforward connections from the LGN^34^,^35^. In contrast, the modulation of spiking activity by attention and working memory was most pronounced in the superficial and deep layers and it was accompanied with strong current sinks in the superficial layers and layer 5, which are the main targets of cortical feedback connections^40^,^41^. Previous studies revealed that input into these layers can boost spiking activity across the cortical column, providing a powerful mechanism of top-down control^33^,^64^. Our findings thereby support the notion that attention and working memory modulate spiking activity in early visual areas through feedback input, although causal experiments would be required to provide conclusive evidence. The laminar pattern of activity during selective attention and working memory resembled the laminar profile when monkeys segregate a texture into figure and background^34^, although figure-ground segmentation also occurs when attention is directed elsewhere^65^. It seems therefore safe to conclude that the sinks in the superficial layers and layer 5 represent a genuine canonical signature of the feedback influences onto V1. Future studies can exploit these distinct laminar profiles of feedforward and feedback influences to further our understanding of the functional impact of feedforward and feedback connections.

## Methods

### Stimuli and task

We trained two male rhesus macaque monkeys (E and R, 12 and 8 years old) to perform the curve-tracing tasks. A trial began with the fixation point (a red circle of 0.3° diameter) presented on a grey background. The monkey initiated the trial when the eye position was within a 1° window centred on the fixation point. We presented the curve-tracing stimulus after 300 ms of fixation and extinguished the fixation point after another 750 ms in most of the tasks (Figs 2a,d, 5a and 6a), but after 1,100 ms in the task with the delayed presentation of the cue near fixation (Fig. 7a). The monkey was required to make a saccadic eye movement into a target-window (3° diameter) centred on the green disc at the end of the target curve. Correct responses were rewarded with apple juice. We aborted trials in which the animal broke fixation before the fixation point was extinguished. All stimulus conditions were presented in a pseudorandom order.

The stimuli were generated using in-house software and were presented on a CRT monitor with a resolution of 1,024 × 768 pixels and refresh rate of 85 Hz, which was viewed from a distance of 75 cm. The stimulus consisted of two main branches that could split into two curves each, with an initial segment (location 1 in Fig. 1a) that determined which of the two main branches was relevant and two additional segments (locations 2 and 3) determining the relevance of contour elements behind this second bifurcation, so that there were a total of 8 stimuli (Supplementary Fig. 1). We placed one of the contour elements at location 2 in the center of the RF. The configuration at location 3 was not relevant for our analyses and we therefore averaged across these two configurations so that four conditions remained.

### Surgical procedures

The animals underwent two surgeries under general anaesthesia that was induced with ketamine (15 mg kg^−1^ injected intramuscularly) and maintained after intubation by ventilation with a mixture of 70% N_2_O and 30% O_2_, supplemented with 0.8% isoflurane, fentanyl (0.005 mg kg^−1^ intravenously) and midazolam (0.5 mg kg^−1^ h^−1^ intravenously). We implanted a head holder in the first operation. We trained the monkeys until they could reliably perform the task, and we then implanted a recording chamber (Crist Instruments) over the operculum of V1 and performed a craniotomy inside the chamber for the laminar recordings. All procedures complied with the NIH Guide for Care and Use of Laboratory Animals (National Institutes of Health, Bethesda, Maryland), and were approved by the institutional animal care and use committee of the Royal Netherlands Academy of Arts and Sciences.

### Data acquisition and preprocessing

We collected neuronal data with Tucker Davis Technology recording equipment using a high-impedance headstage (RA16AC) and a preamplifier (either RA16SD or PZ2) with a hardware high-pass filter of 2.2 Hz, a low-pass filter of 7.5 kHz (−3 dB point) and sampled with a rate of 24.4 kHz. As in previous studies^21^,^66^,^67^, the digitized signals were band-pass filtered (500 Hz–5 kHz), full-wave rectified and low-pass filtered (200 Hz) to produce an envelope of the MUA. This MUA signal provides an average of spiking activity of a number of neurons in the vicinity of the recording site and the population response obtained with this method is therefore expected to be identical to the population response obtained by pooling across single units^66^,^68^,^69^. We applied a low-pass filter (<200 Hz) to record the LFP and sampled it at 763 Hz. We corrected the LFP for the amplitude changes and phase shifts that were induced by the filters in the preamplifier (Supplementary Information).

We used multi-contact ‘U' probes (Plexon) for the laminar recordings with 24 contact points spaced 100 μm apart (Fig. 1f). Either the metal shaft of the probe or a silver/silver chloride wire in the recording chamber served as reference. We used a blunt guide tube to exert slight pressure on the dura and we then quickly inserted the tip of the probe using a micro-manipulator (Narishige, Japan). The moment when the first contact point passed the dura was ascertained by careful visual inspection of the LFP and listening to the MUA. Once the probe passed through the dura, the guide tube and the probe were moved upward until the dura showed no sign of dimpling. This procedure was done quickly to minimize the time of applying pressure to the cortex. The probe was then lowered into the cortex at very low speeds (∼2–5 μm s^−1^). We obtained high quality spiking activity with this method and observed no shifts in the depth of the probe during the recording sessions.

We calculated the one-dimensional CSD from the LFP following Mitzdorf^70^ as:


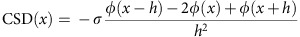


Where *φ* is the voltage, *x* is the point at which the CSD is calculated, *h* is the spacing of electrodes for the computation (here we used a spacing of 200 μm, but we obtained equivalent results with a spacing of 100 μm) and *σ* is the conductivity of cortical tissue (we used a value of 0.4 S m^−1^)^71^. The CSD is negative if currents flow towards the electrode (sink) and positive if currents flow away from the contact points (source)^70^,^72^.

To determine the depth of the electrode we measured the CSD evoked by a full-screen 100% contrast checkerboard (presented for 250 ms while the monkey fixated, check size 0.3°)^34^,^35^,^70^. We estimated the location of the border between layer 5 and layer 4C as the polarity reversal from current sinks in layer 4C to current sources in the deep layers around 40 ms after stimulus onset^35^,^73^. We placed the electrode so that the CSD reversal was as close as possible to the eighth contact from the tip to ensure coverage of all cortical layers. Final alignment was done on the basis of evoked potential by the curve stimuli (Fig. 1i). We mapped the RFs of the neurons at every recording site of the electrode (see below for the RF mapping methods). When the RFs of the recording sites in different layers did not overlap, indicating that the probe was not perpendicular to the cortical surface, the probe was retracted and inserted at a different location. We calculated the signal-to-noise ratios (SNRs) of every MUA recording site as the height of the peak of the stimulus-evoked response divided by the s.d. of activity in the pre-stimulus period. Sites with an SNR<3 were excluded from the analysis. The LFP of excluded channels were replaced by an interpolation of neighbouring channels, to avoid artifacts in the CSD (we excluded <5% of the channels). Eye movements were recorded with a video eye-tracker (Thomas recordings) with a sampling rate of 250 Hz.

### Data analysis

The MUA response at each recording site was normalized by subtracting the spontaneous activity, measured from −150 ms to 0 ms before stimulus onset, and by dividing the response by the peak response (calculated as the maximum response in a window 50–90 ms after stimulus onset) of the recording site in the distractor condition. We normalized the CSD of each penetration by dividing by the maximum absolute value of the CSD across layers during the stimulus period in the target curve condition.

To generate the average MUA and CSD per electrode depth, we first aligned the depths of the different penetrations using the CSD as described above. The realigned, normalized CSD data and normalized MUA signals were then averaged across penetrations. We assigned recording sites to one of three laminar compartments based on their distance from the layer 4C/layer 5 boundary. The assignments were made with reference to previous anatomical studies^74^,^75^, which measured the thickness of the cortical layers in V1. Recording sites between 0.55 and 0.05 mm below the 4C/5-boundary were assigned to the deep layers, sites between 0.05 and 0.55 mm above the boundary were assigned to layer 4 and sites between 0.65 and 1.15 mm above the boundary were assigned to the superficial layers.

Our method to calculate the latency of the visual response was similar to previously described methods^34^. We fitted the sum of a Gaussian and a cumulative Gaussian to the data and determined the latency as the time point at which the fitted curve reached 33% of its maximum.

We quantified the amplitude of the normalized CSD pattern over time (nCSD). To this aim, we first computed the laminar template, defined as the average laminar profile in the modulation period normalized to the channel with the strongest sink. The nCSD is the inner product of the laminar template and the momentary CSD. We also computed the nCSD of the difference between two conditions. To this aim, we average the difference between the conditions over time and use the difference as the laminar template. The absolute value of the modulation of the CSD was calculated by first computing the absolute value of the difference between conditions per channel and averaging these differences across channels.

To calculate the reliability of the MUA and CSD laminar profile within a task we took the average activity in the modulation period and measured the pair-wise correlation across all combinations, that is, 0.5*N*(*N*−1), where *N* is the number of penetrations. For the comparisons between tasks, we compared the same penetration between tasks, that is, the number of correlations equalled *N*. For the shuffle controls, we randomly shuffled the channels from one penetration of the comparison.

We used a support vector machine as input for a receiver operating characteristic (ROC) analysis to determine how well the MUA signal across all layers discriminated between target and distractor curves on a single trial, using the scikit-learn toolbox^76^. We used the average MUA activity per recording site in a window from 200 to 750 ms as input for the support vector machine. Cross-classification was achieved by randomly splitting the trials, using 90% of the trials for the training of the support vector machine and the remaining 10% for testing its output in an ROC analysis, in every recording session. This analysis was repeated 10 times (randomly selecting the 10% of trials for cross-validation) and classification accuracy (from the ROC analysis) was averaged across these 10 repeats. We then averaged these single-session scores across penetrations to obtain the classification accuracy per monkey.

### Receptive-field measurements

We measured the V1 RF dimensions by determining the onset and offset of the response to a slowly moving light bar for each of eight movement directions (Supplementary Fig. 5)^77^. The MUA RF size was 1.4°, on average (with an s.d. of 0.25°), and the eccentricity was between 2.1 and 7.8° (median=5.3°).

### Eye position

Trials containing microsaccades were discarded; microsaccades were defined as a minimum of five consecutive samples in which the speed of the eye movement was higher than five times the s.d. In both monkeys we observed a small bias (<0.05°) for the eye position to shift towards the lower left quadrant, but, importantly, there were no differences in the average eye position or the s.d. of the eye position between conditions (Supplementary Fig. 6).

### Statistics

To estimate the significance of attentional modulation/working memory modulation we calculated statistics at the level of penetrations. Data were averaged across channels per penetration before being entered into a significance test, to correct for the correlations between channels belonging to the same penetration. To compare the MUA across laminar compartments (deep, layer 4 and superficial) we averaged the data across channels within each laminar compartment, and used repeated measures ANOVAs with three laminar compartments as levels. By using a repeated measures design, we corrected for the correlation in activity between laminar compartments of the same penetration. If necessary, we applied the Greenhouse–Geisser correction for deviations from sphericity. We used an analysis based on cluster statistics to determine the reliability of the laminar pattern of the modulation of the CSD by working memory and attention (Supplementary Information).

For post-hoc testing we used Wilcoxon signed-rank test and we used Wilcoxon rank-sum test to compare results from different tasks. The Bonferroni correction was applied in case of multiple comparisons.

### Data availability

The data that support the findings of this study are available from the corresponding authors upon reasonable request.

## Additional information

**How to cite this article:** van Kerkoerle, T. *et al*. Layer-specificity in the effects of attention and working memory on activity in primary visual cortex. *Nat. Commun.* **8,** 13804 doi: 10.1038/ncomms13804 (2017).

**Publisher's note:** Springer Nature remains neutral with regard to jurisdictional claims in published maps and institutional affiliations.

## Supplementary Material

Supplementary Information Supplementary Figures, Supplementary Methods and Supplementary References
