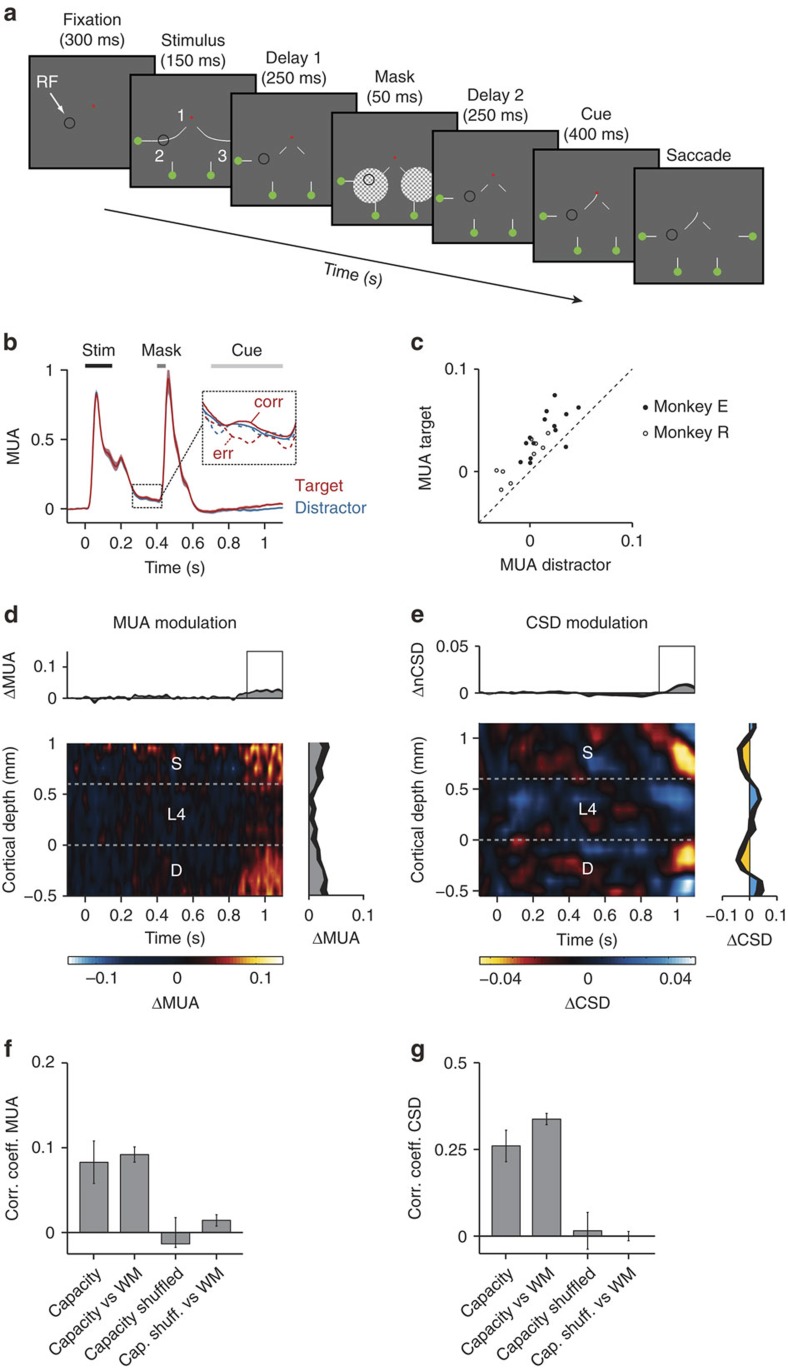# Erratum: Layer-specificity in the effects of attention and working memory on activity in primary visual cortex

**DOI:** 10.1038/ncomms15555

**Published:** 2017-05-03

**Authors:** Timo van Kerkoerle, Matthew W. Self, Pieter R. Roelfsema

Nature Communications 8: Article number: 13804; DOI: 10.1038/ncomms13804 (2017); Published: 01 05 2017; Updated: 05 03 2017

In Fig. 7b of this Article, traces depicting the average MUA response evoked by the target (red) and distractor (blue) were inadvertently omitted during the production process. The correct version of Fig. 7 appears below as Fig. 1.

## Figures and Tables

**Figure 1:**