# Supplementary material for: Layer-specificity in the effects of attention and working memory on activity in primary visual cortex
Source: Nat Commun. 2017 Jan 5;8:13804. doi: 10.1038/ncomms13804 (PMC5227065; doi:10.1038/ncomms13804)
Supplement: Supplementary Information — Supplementary Figures, Supplementary Methods and Supplementary References [file ncomms13804-s1.pdf]

SUPPLEMENTARY FIGURES

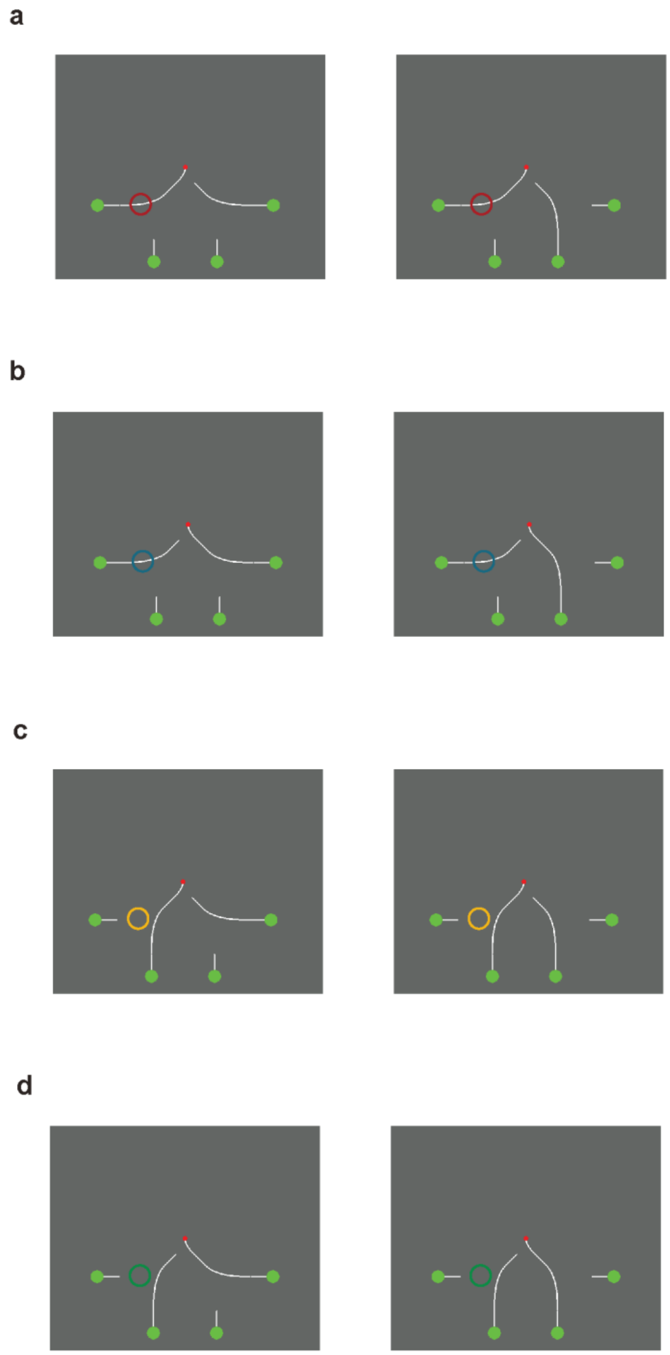

Supplementary Figure 1 | The curve-tracing stimuli. There were two stimuli for each of the conditions, and we averaged neuronal activity across these two stimuli in the analysis. The target (**a**) or distractor curve (**b**) fell in the RF or the target (**c**) or distractor curve (**d**) fell next to the RF. Circles indicate the position of the RFs.

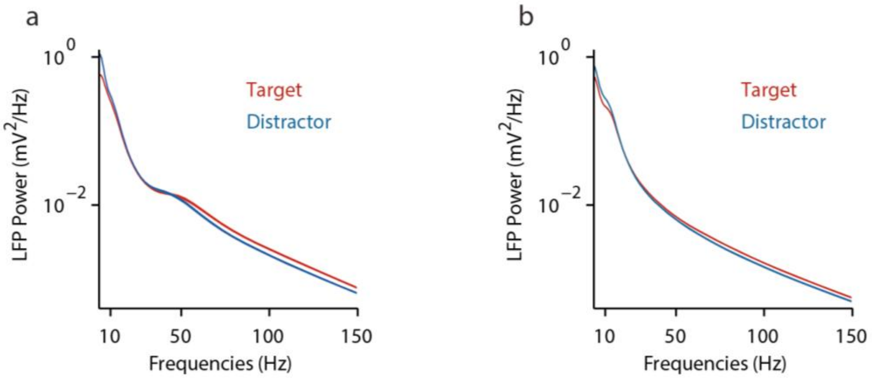

Supplementary Figure 2 | LFP power for attention and working memory. Average LFP power spectrum (mV[2]/Hz) evoked by the target (red trace) and distractor (blue trace) in a time window from 200-750ms after stimulus onset in (**a**) the attention task (Figure 2a), and (**b**) the working memory task (Figure 2d). Shaded areas show s.e.m. (n=37 penetrations; when they are difficult to see the s.e.m. is small).

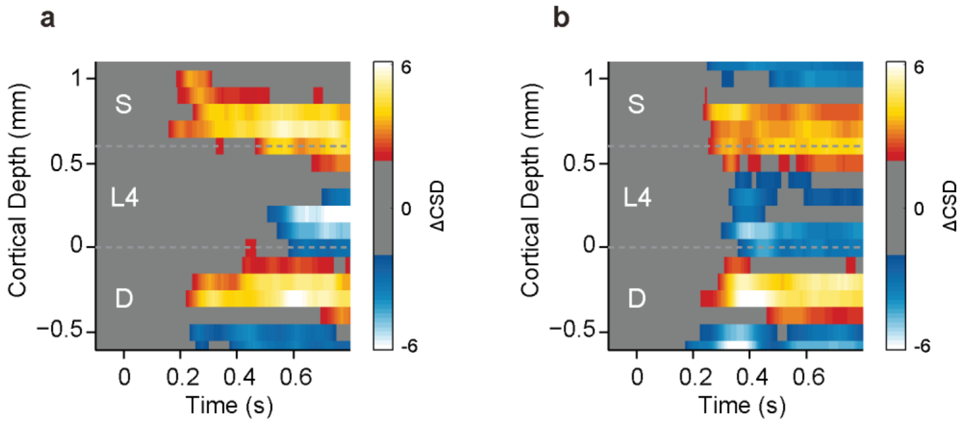

Supplementary Figure 3 | Statistical map of CSD cluster statistics. t-scores of the difference between the CSD evoked by the target and distractor curve in (**a**) the attention task and (**b**) the working memory task. Cluster statistics were used to calculate the significance of sinks and sources. Non-significant clusters are not shown (gray area; p>0.05).

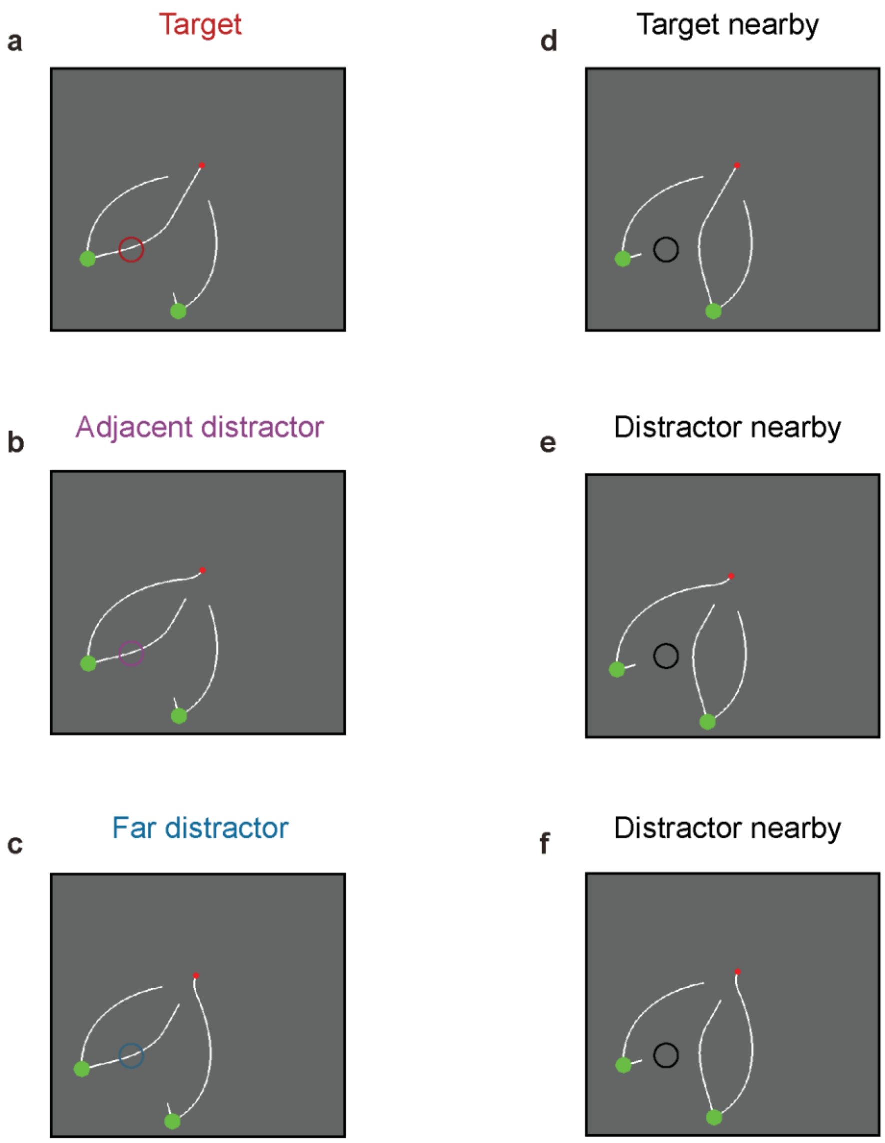

Supplementary Figure 4 | All stimuli of the eye movement control task. The RF could fall on the target (**a**), on the adjacent distractor (**b**) or on the far distractor curve (**c**). There were also three corresponding conditions when the RF fell close to the target (**d**), or close to one of the distractors (**e**, **f**). Circles indicate the position of the RFs.

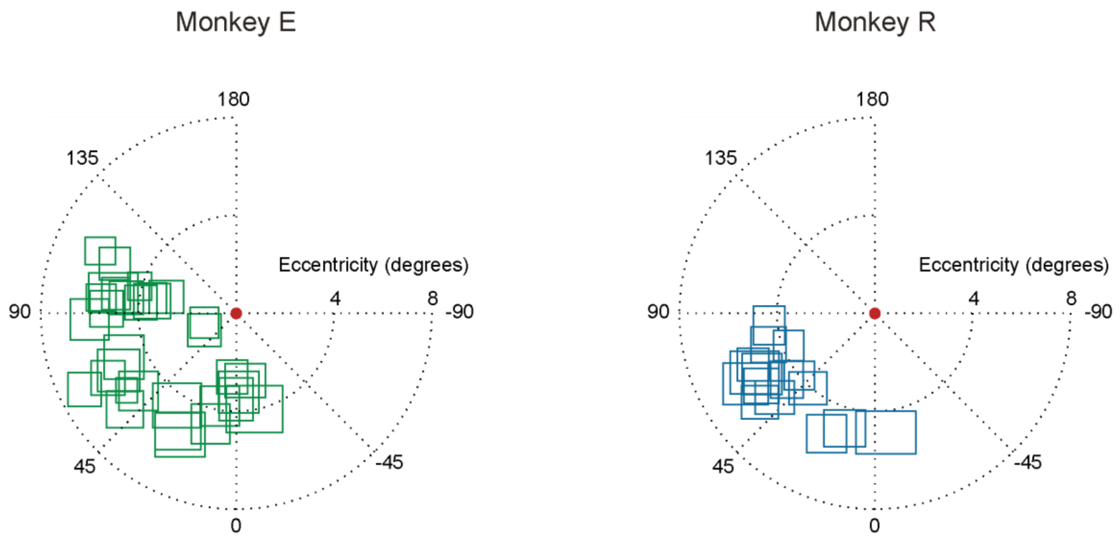

Supplementary Figure 5 | Position and size of the multiunit RFs. The rectangles indicate the average position and size of the RFs in each penetration. Monkey E, green squares. Monkey R, blue squares.

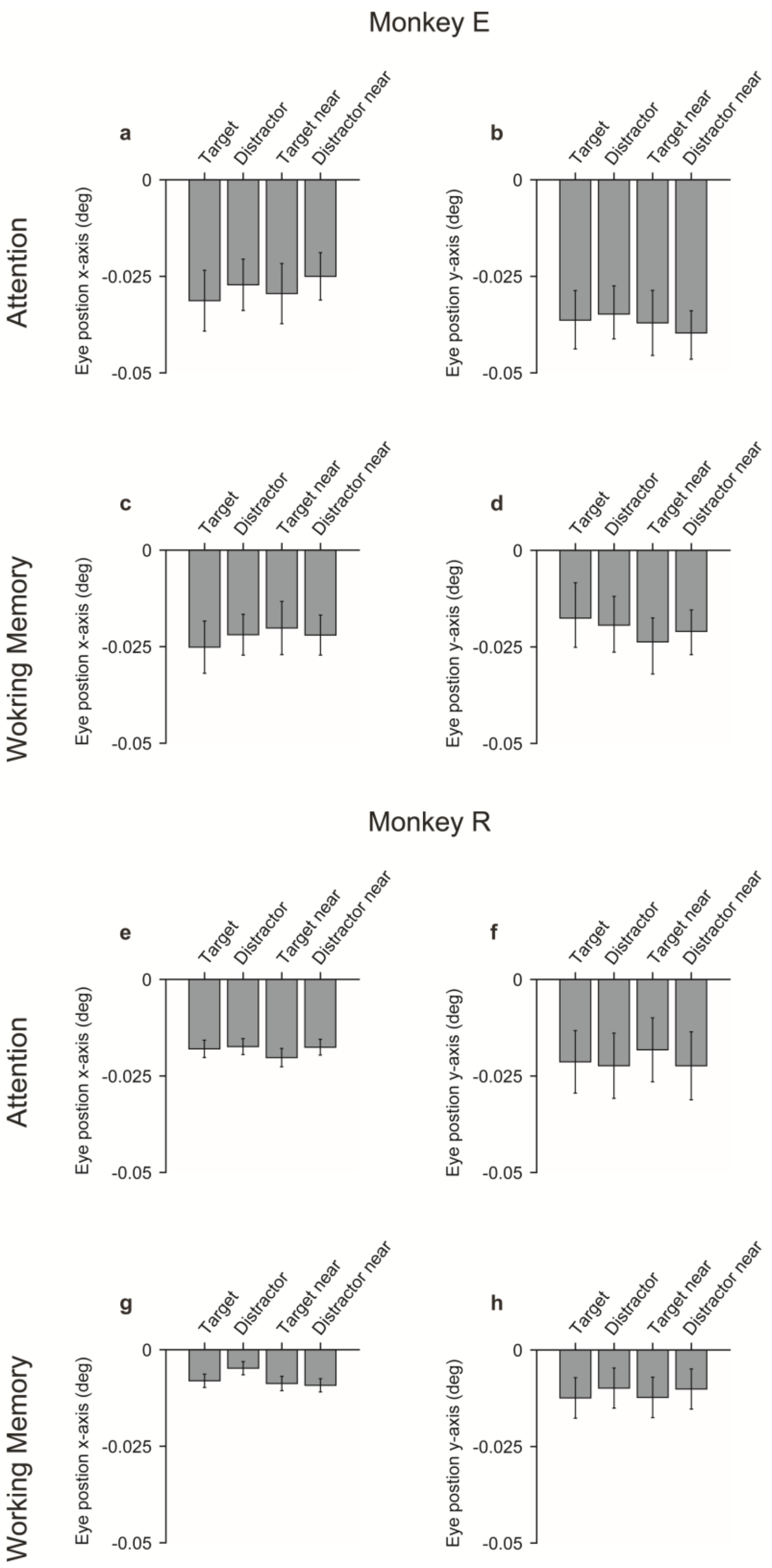

Supplementary Figure 6 | Eye position during the attention and working memory tasks. Horizontal (**a**, **c**, **e**, **g**) and vertical eye position (**b**, **d**, **f**, **h**) in the attention (a, b, e, f) and working memory task (c, d, g, h), for monkey E (a-d) and monkey R (f-h).

SUPPLEMENTARY METHODS

Amplitude and phase correction

We corrected for the change in amplitude and phase shifts induced by the hardware filters[1]. To determine the filter characteristics we generated 15 windows of 60 seconds white noise in Matlab (The MathWorks, Inc), used the sound card of the PC for output and attenuated the signal with a voltage divider. We fed the signal to the headstage and preamplifier, and for comparison also to a DC amplifier (TDTRA8GA). As expected, the power spectrum of the signal recorded with the DC amplifier was flat, while the lower frequencies were attenuated in the signal recorded through the head-stage and preamplifier. We calculated the phase spectrum between the two signals as well as the amplitude spectrum of the regular preamplifier and fitted sigmoidal functions to these spectra. We used the fitted functions to correct LFP signals in the frequency domain.

CSD cluster statistics

The statistical analysis of the significance of differences in the CSD between target and distractor (**Supplementary Figure 3**)[3] was based on a non-parametric cluster analysis[4]. First, we calculated t-scores for the difference in the CSD evoked by the target and distractor curve across penetrations for each time-sample/depth pair, thus producing a 2-dimensional array of t-scores (time-samples x depth). The t-map was thresholded, setting non-significant t-values ($p < 0.05$) to zero. We clustered adjacent t-scores with the same sign and calculated a cluster statistic; the sum of the absolute t-scores per cluster. To determine the significance of these clusters, we carried out a bootstrapping analysis. We randomly shuffled the data (mixing trials from the target and distractor condition) to produce 1000 CSD difference maps and subjected them to the same procedures as the real data. We took the maximum of the sum of the t-values in each cluster as the bootstrap statistic and generated a distribution of this statistic. We then compared the calculated t-values from each cluster of the actual

data to the shuffled distribution. Clusters were considered significant if their absolute t-value fell above the 95th percentile of the shuffled distribution. We also applied a size-threshold, excluding clusters that were smaller than 10 time-sample/depth pairs. The advantage of this statistic is that it uses both the magnitude of the t-scores within a cluster and also the size of the cluster so that is sensitive to strong sinks and sources and also to weak, but spatially extended, sinks and sources.

SUPPLEMENTARY REFERENCES

1. Nelson,M.J., Pouget,P., Nilsen,E.A., Patten,C.D., & Schall,J.D. Review of signal distortion through metal microelectrode recording circuits and filters. *J. Neurosci. Methods***169**, 141-157 (2008).

2. Pedregosa,F. *et al.* Scikit-learn: Machine Learning in Python. *Journal of Machine Learning Research***12**, 2825-2830 (2011).

3. Self,M.W., van Kerkoerle T., Super,H., & Roelfsema,P.R. Distinct roles of the cortical layers of area V1 in figure-ground segregation. *Curr. Biol.***23**, 2121-2129 (2013).

4. Maris,E. & Oostenveld,R. Nonparametric statistical testing of EEG- and MEG-data. *J. Neurosci. Methods***164**, 177-190 (2007).
